# Dentistry and Diabetes: The Influence of Diabetes in Oral Diseases and Dental Treatments

**DOI:** 10.1155/2016/6073190

**Published:** 2016-12-29

**Authors:** Eugenio Velasco-Ortega, Rafael Arcesio Delgado-Ruiz, Jose López-López

**Affiliations:** ^1^University of Seville, Seville, Spain; ^2^Stony Brook University, Stony Brook, NY, USA; ^3^University of Barcelona, Barcelona, Spain

Diabetes is a chronic disease that occurs when the pancreas does not produce enough insulin or when the body cannot effectively use the insulin that it produces. The number of people with diabetes is increasing. These trends highlight the urgency for a better understanding of diabetes as well as for improving the dental care of patients with diabetes. Patients with diabetes have increased frequency of periodontitis, tooth loss, and xerostomia, and diabetes has been considered a risk condition for oral surgery and dental implants with the fact that it is associated with delayed wound healing, prevalence of microvascular disease, and impaired response to infection.

J. Gonzalez-Serrano et al., in a systematic review, showed that a higher prevalence of oral mucosal disorders was found in patients with diabetes mellitus (DM) compared to non-DM patients. This increased prevalence of oral disorders in DM groups may be due to an inadequate metabolic control of DM or a slow healing process.

The results of an experimental study in animals about the oxidative damage caused to the salivary glands in streptozotocin-induced diabetes are presented by M. Knaś et al. and demonstrated that the unstimulated salivary flow in DM rats was reduced in the 2nd week, while the stimulated flow was decreased throughout the duration of the experiment versus control.

R. M. López-Pintor et al., in a systemic review, showed a decreased salivary flow in DM patients in relation to non-DM patients. The reasons for these problems could be due to damage to the gland parenchyma, alterations in the microcirculation to the salivary glands, dehydration, and disturbances in glycemic control.

J. Matczuk et al. observed that the high fat diet regimen had caused significant changes in the salivary glands lipid composition, especially in regard to phospholipids (PH) and triacylglycerol (TG) in rats. The observed reduction in PH concentration is an interesting phenomenon frequently signifying the atrophy and malfunctions in the saliva secreting organs. On the other hand, the increased accumulation of TG in the glands may be an important clinical manifestation of metabolic syndrome and type 2 diabetes mellitus.

The importance of periodontitis in diabetes is assessed by Q. Wang et al. that presented a comparison of experimental periodontitis induced by* Porphyromonas gingivalis* in diabetic mice.

Given the increasing number of dental patients with diabetes worldwide, there is a necessity for a better understanding of mechanisms of the oral manifestations of diabetes and its comprehensive treatment to prevent possible complications.

*Eugenio Velasco-Ortega* *Eugenio Velasco-Ortega* *Rafael Arcesio Delgado-Ruiz* *Rafael Arcesio Delgado-Ruiz* *Jose López-López* *Jose López-López*

